# Long‐Term Pulmonary Sequelae 5–14 Years After Protracted Bacterial Bronchitis in Early Childhood

**DOI:** 10.1002/ppul.71111

**Published:** 2025-05-05

**Authors:** Jan Hermann, Karen Brückner, Cordula Koerner‐Rettberg, Stefanie Dillenhöfer, Folke Brinkmann, Christoph Maier, Christoph M. Heyer, Anne Schlegtendal

**Affiliations:** ^1^ Department of Paediatric Pneumology University Children's Hospital, Ruhr‐University, Bochum Bochum Germany; ^2^ Department of Anesthesiology and Intensive Care Medicine St. Josef and St. Elisabeth‐Hospital, Ruhr‐University Bochum Bochum Germany; ^3^ Department of Paediatrics, Helios University Hospital Wuppertal, University Witten/Herdecke Wuppertal Germany; ^4^ Department of Paediatrics Marien‐Hospital Wesel, Teaching Hospital of University of Münster Wesel Germany; ^5^ Section for Paediatric Pneumology and Allergology University Medical Center Schleswig‐Holstein, Airway Research Center North (ARCN) of the German Center of Lung Research (DZL) Lübeck Germany; ^6^ University Children's Hospital, Ruhr‐University Bochum Bochum Germany; ^7^ Department of Paediatric Pneumology University Children's Hospital, Katholisches Klinikum Bochum, Ruhr‐University Bochum Bochum Germany

**Keywords:** asthma, bronchiectasis, bronchoscopy, children, chronic wet cough, LCI, lung function, nitrogen multiple‐breath washout, pbb

## Abstract

**Background:**

There is little information about long‐term changes in pulmonary function tests (PFTs) many years after protracted bacterial bronchitis (PBB), the most common cause of chronic wet cough in early childhood.

**Methods:**

Of 200 consecutively recruited children with a previously proven diagnosis of PBB 62 (12.2 years, female 48%) were interviewed after 7.7 (5.4–14.7) years about their previous and current symptoms and pulmonary function tests (PFTs: spirometry, body plethysmography, nitrogen multi‐breath washout, exhaled nitric oxide and nasal nitric oxide) were performed. Children with persistent symptoms were offered lung imaging.

**Results:**

11 (17.7%) patients suffered from chronic or recurring wet cough years after their first PBB episode. 15 (24.19%) had at least one abnormal spirometry parameter. FEV1 was abnormal in eight of 62 (12.9%), LCI 2.5% in seven of 56 (12.5%), FVC in 12 of 62 (19.35%) and FEV1/FVC in five of 62 (8.06%) cases. PFT did not differ between children with and without wet cough. Lung MRI/CT demonstrate in four of nine cases abnormalities of the bronchial walls, including one with incipient bronchiectasis.

**Conclusion:**

After PBB in early childhood, a significant proportion of children suffer from respiratory symptoms many years later, some have an objectively reduced lung function and structural changes of the bronchial wall despite adequate initial therapy. Wet cough alone seems not to be a sensitive clinical predictor. Due to the retrospective study design, we cannot proof any causal relationship. However, to detect late bronchopulmonary sequelae, continuous follow‐up of these children should become mandatory.

## 1 Introduction

Cough is the most commonly reported symptom in children and adults, and is the primary reason for consulting a healthcare provider [1, 2, 3]. Chronic cough is defined as frequent coughing for more than 8 weeks in adults or more than 4 weeks in children [4]. While chronic dry cough in childhood is often associated with asthma, protracted bacterial bronchitis (PBB) is the most common cause of chronic wet cough in children, accounting for almost all cases of chronic wet cough in preschool‐age [3, 4, 5, 6]. The burden of chronic cough is high for both, children and their families [7, 8, 9, 10]. PBB was first described in 2006 and is characterized by a wet cough that persists for at least 4 weeks without evidence of a specific cause and resolving after 2–4 weeks of antibiotic treatment [3, 11]. Some of the most common pathogens involved are Haemophilus influenzae, Streptococcus pneumoniae and Moraxella catarrhalis. PBB is characterized by a neutrophilic inflammation of the airways and activation of the innate immune system [11, 12], but the underlying mechanism of PBB is not well understood. PBB is thought to be a major cause of non‐CF bronchiectasis, so early identification of children at risk is a high priority [3, 13].

So far two studies by Wurzel et al. (2016) [13] and Ruffles et al. (2021) [14] have investigated the long‐term effects of PBB in children, with a focus on a potential development of bronchiectasis. Wurzel et al. monitored children over a period of 2 years, performing CT scans on those exhibiting clinical features of bronchiectasis, such as a chronic wet cough unresponsive to 4 weeks of antibiotic treatment, persistent chest radiographic changes despite appropriate oral antibiotic therapy, or recurrent hospitalizations due to acute respiratory events. Ruffles et al. followed their cohort for 5 years. They performed CT scans in accordance with the methods employed by Wurzel et al. Spirometry was carried out on all participants. Both studies revealed a significant proportion of children within the PBB cohort with bronchiectasis (8.1% in Wurzel et al. (2016) and 9.6% in Ruffles et al. (2021)). In both studies an infection with Haemophilus influenzae and recurrent PBB infections were identified as primary risk factors for adverse outcomes. However, Ruffles et al. did not find a statistically significant difference between their cohort and the reference group in terms of forced expiratory volume in the first second (FEV1) or forced vital capacity (FVC). To our knowledge, no previous studies have evaluated the use of a nitrogen multiple breath washout (N_2_MBW) for the assessment of children after PBB.

The aim of our study was to analyze the long‐term effects of PBB in early childhood as measured by pulmonary function testing (PFTs), including spirometry and N_2_MBW, and to determine the accuracy of a history of wet cough in predicting poor outcomes.

## 2 Materials and Methods

### 2.1 Study Design and Patients

**FIGURE 1:**
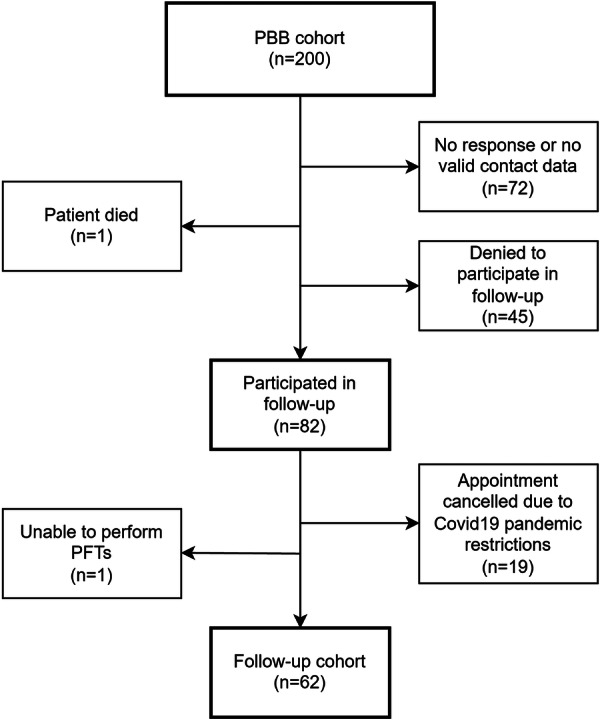
Process‐Flowchart of the study recruitment from the original PBB cohort to the follow‐up cohort. PBB, protracted bacterial bronchitis; PFT, pulmonary function test.

We conducted a prospective single‐center study with a single follow‐up appointment with children and adolescents who were originally treated for PBB in early childhood as inpatients at the University Children's Hospital of the Ruhr‐University Bochum between 2005 and 2014 (PBB cohort). For the PBB diagnosis of the original PBB cohort, the microbiological criteria: (i) history of chronic wet cough > 4 weeks, (ii) BAL with neutrophilia > 20% and/or ≥ 10^4^ colony forming units/ml of potentially pathogenic bacteria like Haemophilus influenzae, Moraxella catarrhalis, Streptococcus pneumoniae or *Staphylococcus aureus*, and (iii) resolution of coughing after antibiotic treatment of at least 14 days, were applied [15]. Other causes of productive cough (e.g., cystic fibrosis, primary ciliary dyskinesia, immunodeficiency) were ruled out in advance. All 200 children of this PBB cohort were contacted and invited to participate in this study (Figure 1). Inclusion criteria were willingness to attend an appointment at the children's hospital, written consent from all adult subjects or parents/guardians of all minor subjects, and the ability to perform PFT tests. The study was approved by the ethics committee of Ruhr‐University Bochum (EC reference no. 18‐6431).

### 2.2 Diagnostics

All participants in the study and their parents were interviewed about their medical history since the initial diagnosis of PBB using a questionnaire that included questions about their history of cough, PBB recurrence or pneumonia, any atopic diseases such as asthma, atopic eczema, or allergies, and possible risk factors for PBB.

Subsequently, all subjects performed pulmonary function tests (PFTs) including body plethysmography, nitrogen multiple‐breath washout (N_2_MBW), exhaled nitric oxide (FeNO) and nasal nitric oxide (nNO) measurements.

Body plethysmography was performed according to the ERS/ATS guidelines using a Masterscreen Body (Vyaire Medical) [16, 17, 18]. The results were classified using the 2012 GLI reference values (2012) [19]. We considered spirometry, body plethysmography and multiple breath washout as abnormal if they were below or above the corresponding lower or upper levels of normal. We considered nNO levels below 200 ppb and FeNO levels over 20 ppb to be abnormal.

N_2_MBW was performed using the Exhalyzer D (Eco Medics AG). The lung clearance index (LCI_2.5%_) conducted by N_2_MBW was initially measured with the Spiroware 3.2.1 software, resulting in falsely high LCI_2.5%_ values because of overestimated N_2_ concentration [20]. A recalculation of the values was carried out after the follow‐up visits with the new Spiroware 3.3.1 software once it was available. The published values of Anagnostopouolou et al. (2020) were used as refence values for LCI_2.5%_ during the follow‐up visits. Once the new reference values for the LCI_2.5%_ measured with the new Spiroware software 3.3.1 were published by Kentgens et al. these were used in the statistical analysis [21]. FeNO and nasal NO measurements were performed with the ANALYZER CLD 88 sp. (Eco Medics AG). All PFTs were done by experienced staff and results were reviewed by experienced pediatric pulmonologists.

As LCI_2.5%_ measured by Multiple Breath Washout is more sensitive than spirometry in detecting structural changes, an elevated LCI_2.5%_ and/or productive cough for more than 8 weeks were considered potential clinical correlates for bronchiectasis [22, 23]. Additionally, each patient case was individually assessed by an experienced pulmonologist for the possible presence of bronchiectasis. Affected subjects were offered additional imaging of the lungs (Magnetic resonance imaging [MRI] or computed tomography [CT]), using the radiologic criteria of broncho‐arterial (BA) ratio > 0.8 to define bronchial dilatation [24]. The appointment was offered as part of the follow‐up study visit. As our center has great expertise in lung MRI and we wanted to minimize the risk of radiation damage, MRI was used wherever possible (MRI scanner: Siemens Magnetom Avanto 1.5 T DOT). All images were acquired with parallel acquisition (iPAT), free‐breathing technique (PACE) and without intravenous contrast (see Supporting Information S1: Table E3 for details about acquired MRI sequences).

Imaging had been offered to some patients based on the initially calculated LCI_2.5%_ values (old software Spiroware 3.2.1), who showed normal values after the later recalculation.

### 2.3 Characterization of Atopic Diseases

The diagnosis of bronchial asthma was doctors‐diagnosed by the pediatric pulmonologists at the study visit based on the medical history, current medication and questionnaires (Asthma control test [25] and questionnaires from the ISAAC study [26]) according to current guideline [27]. A positive atopic history was considered if any allergy‐testing was positive or atopic disease (atopic dermatitis, hay fever, bronchial asthma) was reported in the questionnaire.

### 2.4 Statistical Analysis

Kolmogorov‐Smirnov test was used to evaluate normal distribution. Median and interquartile ranges (IQR) were used for descriptive statistics. We compared continuous variables using the Mann‐Whitney U‐test and used Pearson's chi‐square and logistic regression to assess the correlation between variables. Z‐tests were used to compare the spirometry Z‐scores with the normative cohort from Quanjer et al. (2012) and for the LCI_2.5%_ results with Anagnostopouolou et al. (2020). We considered p‐values less than 0.05 to be statistically significant. Statistical calculations were performed using SPSS statistics (v28.0. 1.1; IBM) and R (v. 4.2.0; R Foundation for Statistical Computing).

## 3 Results

### 3.1 Patient Characteristics

From the original PBB cohort of 200 children (mean age 3.2 years, 51.5% male) with a confirmed diagnosis of PBB between 2005 and 2014, 82 children and their parents were willing to participate in the study and to attend a follow‐up appointment. A total of 62 children (mean age 12.2 years, 51.6% male) could be included (Figure 1), and follow‐up examinations could be performed 7.7 (5–14) years after the initial diagnosis of PBB. There were no significant differences in the main parameters between the follow‐up cohort and the original PBB cohort, apart from a slightly higher average age at diagnosis in the follow‐up cohort (Supporting Information S1:Table E1).

Eleven children (17.7%) reported a chronic, persistent wet cough of at least 4 weeks’ duration at the follow‐up visit or in the previous year. There were significant differences in the burden of disease between the groups with and without a recent history of wet cough: Children with a history of wet cough had higher rates of bronchial asthma, a higher body mass index (BMI) and were more likely to have a history of PBB recurrence or pneumonia (Table 1).

**TABLE 1:** Patient characteristics of the follow‐up cohort at the time of PBB diagnosis and at the time of the follow up visit, organized into sub‐groups by wet cough symptom at follow‐up.

	Total follow‐up cohort	Chronic wet cough a		Chi²	Univariate analysis
		Yes	No	*p*‐value	*p*‐value
n	62	11	51		
male (%)	32 (51.6)	6 (54.5)	26 (51)	0.548	
Follow‐up time, years, median (IQR)	7.7 (6.5–9.9)	9.7 (6.6–12.1)	7.7 (6.4–9.8)		0.185
*At PBB diagnosis*					
Age, years, median (IQR)	4.0 (2.4–5.5)	4.0 (1.3–5.5)	3.9 (2.5–5.5)		0.634
BMI z‐score, median (IQR)	0.36 (–0.4 to 1)	0.7 (–0.2 to 1.6)	0.2 (–0.5 to 0.9)		0.267
History of parental Tobacco exposure (%)	22 (35.5)	6 (54.5)	16 (31.4) b	0.144	
Preterm birth (%)	16 (25.8)	2 (18.2)	14 (27.5)	0.524	
*At follow‐up*					
Age, years, median (IQR)	12.2 (10–14.9)	13.1 (10–15.6)	11.8 (9.9–14.7)		0.404
BMI z‐score, median (IQR)	0.18 (–0.4 to 1.1)	1.2 (–0.3 to 2)	0.1 (–0.5 to 1)		**0.044**
History of PBB relapse (%)	22 (35.5)	8 (72.7)	14 (27.5) b	**0.008**	
History of atopy or positive skin prick test (%)	38 (62.9)	8 (72.7)	30 (58.8)	0.308	
Bronchial asthma, doctor's diagnosed (%)	30 (48.4)	9 (81.8)	21 (41.2)	**0.016**	
History of parental tobacco exposure (%)	14 (22.6)	4 (36.4)	10 (19.6)	0.205	
History of pneumonia, parental report (%)	27 (43.5)	10 (90.9)	25 (49)	**0.011**	

*Note:* Bold type statistically significant (*p* < 0.05).

Abbreviations: BMI, body mass index; Chi², chi‐square test; IQR, Interquartile range; PBB, protracted bacterial bronchitis.

^a^ History of wet cough of at least 4 weeks within 12 months before follow‐up.

^b^ Incomplete data available (*n* = 50).

### 3.2 Pulmonary Function Testing

Spirometry and N_2_MBW showed high proportions of children with abnormal results (Table 2).

**TABLE 2:** Abnormal PFTs according to normative values, tested by binomial test against expected values.

		Abnormal	
		*n*	%
*Spirometry* a	(*n* = 62)	15	24.19
FEV_1_, z‐score		8	12.90
FVC, z‐score		12	19.35
FEV_1_/FVC, z‐score		5	8.06
*N*_*2*_ *MBW*	(*n* = 56)
LCI_2.5%_		7	12.50

Abbreviations: FEV_1_, Forced expiratory volume in the first second; FVC, Forced vital capacity; LCI_2.5%_, Lung clearance index 2.5%; N_2_MBW, Nitrogen multi breath washout.

^a^ At least one abnormal test among FEV_1_, FVC and FEV_1_/FVC.

FEV_1_ and FVC measured by spirometry and LCI_2.5%_ measured by N_2_MBW were statistically significantly more impaired compared to the healthy normative cohort, as determined by the z‐Test of mean (*p* < 0.05).

Of the 15 children with abnormal spirometry results, 60% also had a doctor's diagnosed asthma.

There were no significant differences in the frequencies of abnormal results for the remaining lung function tests (body plethysmography, FeNO and nNO) compared to the normative data.

Comparing children with and without wet cough in the 12 months before follow‐up, we found no difference in lung function. In particular, children with actual productive cough did not have a higher prevalence of lung function abnormalities (Table 3).

**TABLE 3:** Comparison of PFT results between subgroups with and without chronic wet cough.

	Overall		Chronic wet cough a		No chronic wet cough		*U*‐Test
	median	IQR	median	IQR	median	IQR	*p*
*Spirometry*	(*n* = 62)		(*n* = 11)		(*n* = 51)		
FEV_1_, L	2.46	2.1–3	2.61	1.5–4.3	2.39	2.1–2.9	0.712
FEV_1_, z‐score	–**0.32**	–1.1 to 0.3	−0.73	–1.6 to 0	−0.29	–0.7 to 0.5	0.148
FVC, L	2.76	2.1–3.5	2.78	1.8–4.7	2.71	2.1–3.4	0.606
FVC, z‐score	–**0.72**	–1.5 to 0	−0.98	–2 to ‐0.3	−0.61	–1.5 to 0.1	0.242
FEV_1_/FVC, ratio	0.93	0.9–1	0.91	0.8–1	0.94	0.9–1	0.512
FEV_1_/FVC, z‐score	0.98	–0.3 to 2.1	0.55	–1 to 2.1	1.00	–0.2 to 2.2	0.467
*N*_*2*_ *MBW*	(*n* = 56)		(*n* = 10)		(*n* = 46)		
LCI_2.5%_	5.87	5.5–6.3	5.97	5.3–7.6	5.81	5.5–6.1	0.454
*FeNO*	(*n* = 61)	(*n* = 10)	(*n* = 51)				
ppb	8.80	6.8–13	9.3	6–13.1	8.80	7.2–12.9	0.815
*nNO*	(*n* = 62)		(*n* = 11)		(*n* = 51)		
ppb	683.30	575.7–931.8	856.4	518.2–943.6	677.30	576–929.3	0.612

*Note:* Bold type statistically significant in *z*‐Test.

Abbreviations: FeNO, fractional exhaled nitric oxide; FEV_1_, Forced expiratory volume in the first second; FVC, Forced vital capacity; IQR, Interquartile range; LCI_2.5%_, Lung clearance index 2.5%; nNO, nasal nitric oxide; N_2_MBW, Nitrogen multi breath washout; ppb, parts per billion.

^a^ History of wet cough of at least 4 weeks within 12 months before follow‐up.

### 3.3 Lung Imaging

12 subjects were offered further diagnostic lung imaging. Eight patients underwent MRI scan and one patient underwent CT scan. Four patients showed bronchial wall thickening on imaging, one of whom had incipient bronchiectasis (Supporting Information S1: Table E2). Three of these four children with abnormal imaging also had a doctor's diagnosed asthma.

## 4 Discussion

To date, two studies have investigated the long‐term effects of PBB on the respiratory tract [13, 14]. To the best of our knowledge, the study presented here is the first European long‐term follow‐up study to include lung function measurements in children and adolescents with confirmed PBB in early childhood. Our results basically confirm the alarming outcomes of the Australian studies. Despite adequate antibiotic treatment of PBB in early childhood, a meaningful proportion of children and adolescents suffer from respiratory symptoms 5 to 14 years later, such as wet cough and bronchial asthma, in almost half of the children. It is also noteworthy that almost a quarter of the children had pathological spirometry measurements (24%) or an abnormally high LCI_2.5%_ value (12.5%). Four out of nine children examined by CT or MRI showed abnormal bronchial wall changes, in one case even bronchiectasis. Wurzel et al. (2016) [13] and Ruffles et al. (2021) [14] were the first to demonstrate the high proportion of children in two Australian cohorts with persistent symptoms and long‐term sequelae even several years after the initial PBB episode. Our work confirms these results and shows that their findings are replicable in a European cohort. 17.7% of our children suffered from episodes of chronic wet cough, and even 35.5% reported recurrent episodes of PBB, pointing to the disease's enormous long‐term impact, even after many years, which is even higher than the rate of 12.9% in Ruffles et al.

Only Ruffles et al. in 2021 had investigated lung health in a PBB cohort using only spirometry so far. While their findings did not reveal any notable deviations from normal, in our cohort, 27% of children showed signs of lung function impairment in spirometry and/or N_2_MBW. The longer follow‐up period in our study, over the 5 years Ruffles et al. did, may explain this difference.

In other chronic productive lung diseases, the measurement of LCI_2.5%_ is considered a sensitive parameter for detecting lung function decline [28, 29, 30]. Our study also found evidence of this in children and adolescents after PBB in early childhood, so that LCI_2.5%_ might be a valuable tool in the follow‐up diagnostics after PBB.

As in our study, a high rate of asthma in PBB patients has been described in the literature. Donnelly et al. (2007) [31] reported that 31% of their patients with PBB had a firm diagnosis of concomitant asthma in their retrospective cohort. Ruffles et al. (2021) reported an asthma rate of 27.1% in their post‐PBB cohort. In our cohort, almost one in two reported having bronchial asthma, and particularly the subgroup with chronic wet cough had an asthma rate of 81.8%. This high proportion of children with bronchial asthma in our cohort is also much higher than the 6% found in the KiGGS study for the average German population aged 0–17 years [32]. This higher asthma rate cannot be explained by our data. The longer observation period compared to other studies could explain this. Nevertheless, the risk for bronchial asthma seems to be increased for children after one or more episodes of PBB.

Ruffles et al. (2021) found bronchiectasis in 9.6% and Wurzel et al. (2016) in 8.1% of cases. We found structural changes such as bronchial wall thickening in four out of nine patients, one of whom had already developed bronchiectasis. We did not detect any clinical predictor of the abnormalities described above. The lung function in children with a history of or current wet cough at follow‐up was not worse than the lung function in children without a recent wet cough.

### 4.1 Strengths and Limitations

The main limitation of our study is its retrospective design. Therefore, we cannot prove a causal relationship between current symptoms and findings and previous PBB, however plausible this may seem. As we did not have access to a variety of data on children's medical history and as we relied on self‐report or parental reports of past symptoms without a history of clinical assessment, such as regular lung function tests, a recall bias cannot be ruled out.

Other limitations of our study include its single‐center design and the lack of a control group. Due to the restrictions of the SARS‐CoV‐2 pandemic, we had to stop recruitment early and were only able to enroll a relatively small number of cases. Compared with larger cohorts and other PBB cohorts, the proportion of preterm infants and exposure to parental smoking were higher in our PBB cohort [14, 33]. This might be a confounder.

As the inclusion of PBB cases was limited to bronchoscopically proven PBB patients only, the results are not fully generalizable to larger PBB populations. However, due to our strict inclusion criteria, we can be sure to only include patients with a confirmed PBB diagnosis, which is one of the strengths of our study. Another strength is the long follow‐up period.

It cannot be ruled out that there was a bias in favor of patients who still had health problems, so they were more likely to participate in the follow‐up, which means that our prevalence figures in the overall population may be lower. However, the follow‐up cohort is representative of the original PBB cohort, as it does not differ significantly in the main characteristics. Due to ethical restrictions on radiation exposure, not all participants received a CT scan, so it is possible that not all structural late effects were recorded. As the targeted antibiotic treatment was started in the hospital and the subjects were discharged after the initial response with a recommendation for the duration of therapy while still being treated, we have no information on possible treatment failure or early discontinuation of therapy by the local pediatrician after discharge.

## 5 Conclusions

Our study indicates that a significant proportion of children with PBB in early childhood suffer from respiratory symptoms many years later and that almost a quarter of them have objectively reduced lung function. A number of children show changes in the bronchial wall, which can be an early sign of later bronchiectasis. Unfortunately, we found no clinical predictor of this risk, such as a wet cough. Due to the retrospective design of our study, a causal relationship between these changes and PBB cannot be established. This would require multicentre cohort studies. However, the association is clinically and pathophysiologically plausible. It is therefore strongly recommended that children with PBB in early childhood be followed closely to intervene as early as possible. This is because PBB is clearly not a disease that ends with appropriate therapy in childhood but may be the starting point for a lifelong disorder.

## Author Contributions

**Jan Hermann:** conceptualization, methodology, formal analysis, writing – original draft, writing – review and editing, data curation, investigation, validation, visualization. **Karen Brückner:** writing – review and editing, data curation, investigation, validation. **Cordula Koerner‐Rettberg:** conceptualization, methodology, formal analysis, writing – review and editing, supervision. **Stefanie Dillenhöfer:** writing – review and editing, data curation, investigation, validation. **Folke Brinkmann:** conceptualization, methodology, formal analysis, writing – review and editing. **Christoph Maier:** conceptualization, methodology, formal analysis, writing – review and editing, supervision, data curation, investigation, validation. **Christoph M. Heyer:** resources. **Anne Schlegtendal:** conceptualization, methodology, formal analysis, writing – original draft, writing – review and editing, supervision, data curation, investigation, validation, visualization.

## Ethics Statement

By the Ethics Committee of Ruhr‐University Bochum (EC reference No. 18‐6431).

## Conflicts of Interest

The authors declare no conflicts of interest.

## Supporting information

E‐Supplement.

## Data Availability

The data that support the findings of this study are available in the Supporting material of this article or are available from the corresponding author upon reasonable request.
