# Supplementary material for: Long‐Term Pulmonary Sequelae 5–14 Years After Protracted Bacterial Bronchitis in Early Childhood
Source: Pediatr Pulmonol. 2025 May 5;60(5):e71111. doi: 10.1002/ppul.71111 (PMC12053102; doi:10.1002/ppul.71111)
Supplement: Supplementary file 1 — E‐Supplement. [file PPUL-60-0-s001.docx]

Demographic and diagnostic data of the PBB and follow-up cohort at the time of PBB diagnosis - Table E1

|  |  |  | PBB cohort | follow-up cohort |
| --- | --- | --- | --- | --- |
| n | | | 200 | 62 |
| Male, n (%) | | | 103 (51.5) | 32 (51.6) |
| Age, years, median (IQR) | | | **3.2 (1.9-4.6)** | **4.0 (2.4-5.5)** |
| BMI, median (IQR) | | | 16 (15.1-17.2) | 16.3 (15.5-17.3) |
| Tobacco exposure, n (%) | | | 87 (43.5) | 22 (35.5) |
| Bronchial malformation in bronchoscopy, n (%) | | | 36 (18) | 10 (16.1) |
| *Stenosis, n* | | | *30* | *9* |
| *Malacia, n* | | | *2* | *1* |
| *Others, n ^a^* | | | *4* | *0* |
| BAL bacterial growth, n (%) ^b^ | | | 156 (78) | 48 (77) |
| *Haemophilus influenzae, n* | | | *107* | *31* |
| *Streptococcus pneumoniae, n* | | | *24* | *10* |
| *Staphylococcus aureus, n* | | | *20* | *8* |
| *Moraxella catarrhalis, n* | | | *14* | *3* |
| *Pseudomonas aeruginosa, n* | | | *7* | *3* |
| *Others, n ^c^* | | | *28* | *13* |
| Neutrophil count %, mean (SD) | | | 57 (35-80) | 50 (26-73) |
| Antibiotic treatment days, mean ^d^ | | | 21.25 | 19.76 |

Abbr.: PBB: protracted bacterial bronchitis; IQR: Interquartile range; BAL: bronchoalveolar lavage
^a^ Others contained: atypical anatomy, blind ends
^b^ in some subjects there was evidence of multiple bacteria
^c^ Others contained: S. pyogenes, M. pneumoniae, Clamydophilia pneumoniae, Enterococci, K. pneumoniae, Serratia marcesens, E. coli, Porphyromonas, S. epidermidis, E. aerogenes, Citrobacter
^d^ The treatment was started inpatient and the duration and was suggested to the attending pediatrician
Bold type: statistically significant difference in t-test (p < .05)

Pathological chest imaging results of 4 children in the follow-up after protracted bacterial infection in infancy - Table E2

|  | | |
| --- | --- | --- |
|  |  |  |
| MRI 1 |  | String-like, subsegmental hypoventilation in the anterior segment of the right upper lobe. Additionally, signs of chronic deforming bronchitis of the segmental and subsegmental bronchi in both lower lobes without clear bronchiectasis. No mucus plugging, no extensive pneumonic infiltrates. The remaining lung tissue and mediastinal structures are normal. No pathologically enlarged lymph nodes, no other suspicious lesions for tumors. No vascular anomalies. |
| MRI 2 |  | MRI findings indicate a completely unremarkable and age-appropriate condition of the lung tissue without evidence of consolidating infiltrates or significant ventilation disturbances. There is only a slight thickening of the walls of the subsegmental and segmental bronchi in both lower lobes, consistent with chronic bronchitis, without evidence of bronchiectasis. No mucus retention. Pleural structures are unremarkable, no effusion. The mediastinum and lung hila are also normally configured. No vascular anomalies. No pathologically enlarged mediastinal or hilar lymph nodes. The chest wall is intact. |
| MRI 3 |  | Symmetrical and unremarkable lung ventilation without evidence of consolidating infiltrates or significant ventilation disturbances. No bullous or solid intrapulmonary lesions. Mild wall irregularities of the segmental and subsegmental bronchi in both lower lobes, consistent with chronic bronchitis, without evidence of bronchiectasis. Pleural structures are unremarkable, no effusion. The mediastinum and lung hila are also normally configured. Remnant thymus in the anterior upper mediastinum. No pathologically enlarged mediastinal or hilar lymph nodes. No other signs of space-occupying lesions. The mediastinal vascular situation is unremarkable. The chest wall is intact. The upper abdominal organs included in the scan are also unremarkable. Signs of basally accentuated chronic bronchitis with otherwise age-appropriate unremarkable MRI findings of the thoracic organs. No pneumonic infiltrates, no bronchiectasis. Mediastinal structures are also normal. |
| CT 1 |  | Chronic partial atelectasis of the right middle lobe and the laterobasal segment of the left lower lobe, with some pleurodiaphragmatic adhesions as post-inflammatory residues. Bronchiectasis beginning in the right middle lobe, the rest of the lung has a maximum of chronic deforming bronchitis. The remaining lung tissue is currently unremarkable. No active pneumonia, no interstitial lesions. Mediastinal structures are also unremarkable. No pathologically enlarged lymph nodes, no other suspicious or active inflammatory changes. |

All report-texts have been translated from German

MRI Sequences used for detection of bronchiectasis - Table E3

|  | | |
| --- | --- | --- |
|  |  |  |
| MRI scanner |  | SIEMENS Magnetom Avanto 1.5 T DOT |
| MRI sequences |  | All images were acquired with parallel acquisition (iPAT) and free-breathing technique (PACE)    T2w HASTE (Half-Fourier Acquisition Single-shot Turbo spin Echo) in transverse and coronar plane (TR 589 ms, TE 36 ms)  T2w BLADE (motion insensitive, multi-shot Turbo Spin Echo) in transverse and coronar plane  T1w TSE in transverse and coronar plane (TR 778 ms, TE 7.2 ms)  T1w flash 2D in transverse and coronar plane (TR 211 ms, TE 4.76 ms)  T2w TRUFI (True Fast Imaging with Steady-State Free Precession) in transverse and coronar plane (TR 482 ms, TE 1.4 ms)    No intravenous contrast was administered |
